# Supplementary material for: Role of multi-layer tissue composition of musculoskeletal extremities for prediction of in vivo surface indentation response and layer deformations
Source: PLoS One. 2023 Apr 21;18(4):e0284721. doi: 10.1371/journal.pone.0284721 (PMC10121013; doi:10.1371/journal.pone.0284721)
Supplement: Attachment — Submitted filename: reviewer_response.docx [file pone.0284721.s001.docx]

Response to the Reviewer:

We thank the reviewer for their valuable feedback. Responses to the comments have been included below, with reference line numbers in the marked up manuscript.

*Reviewer Specific Comments to the Corresponding Author:*

*1. The current introduction section provides limited background information, please further highlight the research necessity and potential value. Why is it important to determine the effects of individual soft tissue layer architecture on the mechanical behavior of multi-layer tissue regions across different sites within human in-vivo extremities.*

Author Response:

The introduction section was expanded with some of the research that justifies the statistical modeling effort. Several additional citations and research avenues were added in lines 44-49. Surgical simulation represents one of the most intriguing areas (lines 52-54). Justification on why statistical models were used over traditional physics simulations like finite element analysis was also added to the introduction.

*2. What was the basis for selecting the eight sites of the upper and lower extremities?*

Author Response:

The paper was revised to justify why the 8 different locations were selected in lines 95-101. While anatomical measurements were performed in reference 20 at 48 different locations to obtain tissue thicknesses across the musculoskeletal extremities, the central region of each extremity was chosen given its distance from the bone. Proximity to an extremity bone, such as a knee, made reproducible stiffness measurements more challenging.

*3. The methodology involving technical subjects (especially physics inspired statistical models) is difficult to understand, please provide more details to make it clearer.*

Author Response:

The physics inspired statistical model description was updated in lines 134-162. Greater effort was put in justifying the physics rational of the model. Further explanation for Equation 1, 2, and 3 was provided. Further justification was provided on why the model was forced through a 0 y-intercept in lines 159-163. A sentence was added into the statistical tests section to provide a quick explanation on how linear mixed effect models function (lines 129-131).

*4. ‘…that the upper leg region is very likely to have a thicker layer of muscle than the lower arm region for most individuals.’, please add a ref here.*

Author Response:

A citation from a previous study has been added to support this point with reference 27 in the updated bibliography. Muscle in the thigh tends to outweigh muscle in the arm, and the lower extremities exceed the upper extremities by partial volume. Lines 260-263 have been updated with this study’s work.

*5. In summary, please ensure that your manuscript is prepared correctly (without any grammatical and spelling mistakes) and formatted before submitting a revision.*

Author Response:

Various edits and corrections were made throughout the paper to improve the paper outside the above comments, such as removing first person language.
