# Supplementary material for: Role of multi-layer tissue composition of musculoskeletal extremities for prediction of in vivo surface indentation response and layer deformations
Source: PLoS One. 2023 Apr 21;18(4):e0284721. doi: 10.1371/journal.pone.0284721 (PMC10121013; doi:10.1371/journal.pone.0284721)
Supplement: Attachment — Submitted filename: second_reviewer_response.docx [file pone.0284721.s002.docx]

Response to the Reviewer:

We thank the reviewer for their valuable feedback. Responses to the comments have been included below, with reference line numbers in the marked up manuscript.

*Reviewer Specific Comments to the Corresponding Author:*

*1. This manuscript examines how the soft tissue architecture contributed to the aggregate mechanical surface response across 8 different sites of the upper and lower extremities. There is a great insight into investigating the soft tissue. However, the abstract section and the results section mentioned that ‘the fat layer deformation was predicted best by the combined locations model, while the muscle layer deformation was predicted best by the location-specific model’, it’s not in line with the purpose presented in the paper, and the reviewer is confused as to whether the authors intend to compare the prediction effects of the two prediction models on soft tissue deformation or to prove how the soft tissue architecture contributed to the aggregate mechanical surface response across 8 different sites of the upper and lower extremities? The title and purpose were more inclined to explore the role of multi-layer tissue composition of musculoskeletal extremities for the prediction of in vivo surface indentation response and layer deformations. But in the suggestion section, the results are more inclined to compare the difference between the two prediction models.*

Author Response:

A new paragraph was added at the beginning of the discussion to more closely describe the interplay between layers. The discussion section was reworked to make it more clear how the discussion section was related to the two hypotheses. The abstract was also reworked for improved paper clarity.

*2. The methods section of this manuscript is more important, the deformation simulation of soft tissue is an important part of calculated results, but the existing deformation calculation methods and the physical models are difficult to achieve high accuracy and real-time performance. Therefore, the author how to make sure the accuracy and applicability of the calculation method in the article? Is there any relevant literature reference?*

Author Response:

The accuracy of the calculation method can be shown in Figure 4 and 5, showing the Physics based model has an error of around 25-30%. This was discussed in the newly added first discussion paragraph (lines 284-304). Whether this is sufficiently high accuracy would depend on the application of the models. These models requires only simple annotations of ultrasound for tissue thicknesses, which makes them usable without force transducers or camera systems. The models are probably less accurate than some complex models that exist in literature, but require only simple inputs and no parameter search. The value of the models depends on the scenario, which has been added in lines 291-301.

*3. The complex mechanical properties of human soft tissue structure are closely related to physiological and pathological states, so it is necessary to establish and adopt a suitable constitutive model to describe the deformation behavior of soft tissue in mechanical imaging. But the construction of this model is not mentioned in the methods section of the author. Please descript it in detail.*

Author Response:

This model is phenomenological spring based representation of layers. If this work used finite element analysis, it would need a constitutive model. Additionally, tissue behavior was approximated to springs in series rather than being modeled with a constitutive model such as the Mooney-Rivlin model due to a lack of time related data, such as rate dependency and accurate strain measurements across deformation. Spring based modeling is more common in mesh based applications [1], gait [2,3], or simplified modeling of extremities [4]. A benefit of this simplification is that a single metric for stiffness may allow for simpler comparison between regions and subjects as [1] and [2] note the benefit of simplification on providing understanding. Hyperelastic materials also still behave like linear elastic materials under small loads. The above response has been more clearly explained in several portions of the paper, in lines 59-66, 177-179, 299-303.

*4. “two hypotheses were investigated to meet this goal”, but in the suggestion section, the reviewer did not see the author make a detailed discussion on hypothesis 2.*

Author Response:

The discussion was reformatted to better align the discussion with the two hypotheses.

*5. Fat tends to be over-predicted for the combined locations model suggesting the underprediction of muscle deformation is compensated by an over-prediction for fat deformation. Can specific analyses or quantitative data be used to demonstrate the interaction between fat and muscle deformation? Or provide relevant reference documentation.*

Author Response:

This is what we are suspecting, based on the spring based coefficients. Because the problem was formulated as an optimization to aggregate surface response, many combinations of different spring stiffnesses are possible. The springs could be fit individually, but that requires annotation of the different layers at all times.

*6. As the author mentioned in the limitation part, there are big problems in the current research method, which is also the concern of the reviewer.*

Author Response:

Some of the limitations of this work, such as a lack of inclusion of demographic related parameters in the model are being addressed in future models built. Limitations such as lack of resolution to determine skin thickness are not addressable unless different ultrasound equipment is used, which may not be feasible in a hospital setting. The standard deviations for skin was greater than the actual measurement in this dataset, so its inclusion in a model would be just fitting to noise [5]. 25% accuracy may be sufficient depending on application.

*7. All Figure’s clarity is not enough, recommended to improve their quality. The format of the Table is wrong. There are many handwriting errors in the manuscript, such as too many spaces. Please check carefully. Figure 1 needs more comments to explain.*

Author Response:

Figures were formatted with the PACE editor provided by PLOS ONE. The authors also note that the PLOS ONE compiled submission PDF includes low-resolution preview images of the figures after the reference list. The function of these previews is to allow you to download the entire submission as quickly as possible. Click the link at the top of each preview page to download a high-resolution version of each figure. Figure 1 was elaborated upon in Lines 135-139.Tables were reformatted to include all non-title elements inside the table legend. Typographical errors in the manuscript were fixed in the resubmission and the authors thank the reviewer for pointing them out.

Bibliography:

1.	Kähler K, Haber J, Seidel H-P. Geometry-based Muscle Modeling for Facial Animation.

2.	Passive Dynamic Walking - Tad McGeer, 1990. Available: https://journals.sagepub.com/doi/abs/10.1177/027836499000900206

3.	Whittington BR, Thelen DG. A Simple Mass-Spring Model with Roller Feet can induce the Ground Reactions Observed in Human Walking. J Biomech Eng. 2009;131: 011013. doi:10.1115/1.3005147

4.	Butler RJ, Crowell HP, Davis IM. Lower extremity stiffness: implications for performance and injury. Clin Biomech. 2003;18: 511–517. doi:10.1016/S0268-0033(03)00071-8

5.	Neumann EE, Owings TM, Schimmoeller T, Nagle TF, Colbrunn RW, Landis B, et al. Reference data on thickness and mechanics of tissue layers and anthropometry of musculoskeletal extremities. Sci Data. 2018;5: 180193. doi:10.1038/sdata.2018.193
